# CD19+ B cell subsets in the peripheral blood and skin lesions of psoriasis patients and their correlations with disease severity

**DOI:** 10.1590/1414-431X20165374

**Published:** 2016-08-15

**Authors:** J. Lu, Y. Ding, X. Yi, J. Zheng

**Affiliations:** 1 Shanghai Skin Diseases Hospital, Shanghai, China; 2 Department of Dermatology, Ruijin Hospital, Shanghai Jiaotong University School of Medicine, Shanghai, China

**Keywords:** Psoriasis, B-lymphocytes, B-lymphocyte subsets, Psoriasis Area Severity Index (PASI), Flow cytometry

## Abstract

T lymphocytes are important in the pathogenesis of psoriasis, and increasing evidence indicates that B cells also play an important role. The mechanisms of action, however, remain unclear. We evaluated the ratios of CD19+ B cells in peripheral blood mononuclear cells (PBMCs) from 157 patients with psoriasis (65 patients with psoriasis vulgaris, 32 patients with erythrodermic psoriasis, 30 patients with arthropathic psoriasis, and 30 patients with pustular psoriasis) and 35 healthy controls (HCs). Ratios of CD19+ B cells in skin lesions were compared with non-lesions in 7 erythrodermic psoriasis patients. The Psoriasis Area Severity Index (PASI) was used to measure disease severity. CD19+ B cell ratios in PBMCs from psoriasis vulgaris (at both the active and stationary stage) and arthropathic psoriasis patients were higher compared with HCs (P<0.01), but ratios were lower in erythrodermic and pustular psoriasis patients (P*<*0.01). CD19+ B cell ratios in erythrodermic psoriasis skin lesions were higher than in non-lesion areas (P<0.001). Different subsets of CD19+CD40+, CD19+CD44+, CD19+CD80+, CD19+CD86+, CD19+CD11b+, and CD19+HLA-DR+ B cells in PBMCs were observed in different psoriasis clinical subtypes. PASI scores were positively correlated with CD19+ B cell ratios in psoriasis vulgaris and arthropathic psoriasis cases (r=0.871 and r=0.692, respectively, P*<*0.01), but were negatively correlated in pustular psoriasis (r=-0.569, P<0.01). The results indicated that similar to T cells, B cells activation may also play important roles in different pathological stages of psoriasis.

## Introduction

Psoriasis is a chronic and recurrent autoimmune inflammatory skin disease, and there are strong evidences that T lymphocytes are important in its pathogenesis (1). For example, CD4+ T cell and T helper 17 cell levels are significantly elevated in the peripheral blood of psoriasis patients, and large γδ T cell infiltration has been observed in psoriasis skin lesions (2–7). However, other immune cells besides T cells, such as B lymphocytes, macrophages, monocytes, neutrophils, and natural killer (NK) cell levels are also increased in psoriasis patients, and therefore they may contribute to its pathogenesis (8). As evidence of B cell influence in psoriasis pathogenesis, elevated levels of interleukin (IL)-21 were found in the skin lesions of patients with psoriasis (8). IL-21 acts on B cells, resulting in their proliferation, maturation, and transformation into memory B cells (9). Moreover, B cells can help promote CD4+ T cells, and have the potential to produce IL-17, thereby contributing to the inflammation in psoriasis (10). On the other hand, CD19+CD24hiCD38hi B cells secrete IL-10 (11), which has a protective effect on psoriasis (12). However, the exact role of B cells in the pathogenesis of psoriasis remains unclear.

Analysis of cell surface markers expression on B cells (i.e., CD40, CD44, CD80, CD86, CD11b, and HLA-DR) is the key to understand their pathogenic role in psoriasis. CD40 is a surface antigen related to T and B cell function, and CD44 has previously been shown to be upregulated in peripheral blood mononuclear cells (PBMCs) from psoriasis vulgaris patients compared to non-lesional areas (13). Moreover, CD80 and CD86 are significantly increased in PBMCs from psoriasis vulgaris patients than in healthy people, and their costimulatory abnormalities may play a role in the pathogenesis of psoriasis (14,15). CD11b is a main surface receptor for cell adhesion, and its expression can be used as a monitoring index of pustular psoriasis activity (16). Finally, PBMCs can express major histocompatibility class II (MHC II) molecules, such as HLA-DR, thereby becoming antigen-presenting cells, which may have a synergistic effect on the activated T cells in psoriasis.

In this study, we used a case-control approach to examine the ratios of CD19+ B cell subsets (i.e., CD19+CD40+, CD19+CD44+, CD19+CD80+, CD19+CD86+, CD19+CD11b+, and CD19+HLA-DR+ B cells) in PBMCs from psoriasis patients with the vulgaris, erythrodermic, pustular, and arthropathic clinical subtypes, as well as in the skin lesions and non-lesion areas of 7 erythrodermic psoriasis patients. Moreover, we determined whether these subsets of B cells are correlated with psoriasis severity.

## Material and Methods

### Participants

Patients were selected from September 2013 to January 2015 from the Shanghai Skin Diseases Hospital (China), including patients from Shanghai and neighboring cities. Inclusion criteria were as follows: a) clinical diagnosis of psoriasis vulgaris, erythrodermic psoriasis, arthropathic psoriasis, or pustular psoriasis (palm and plantar only) (17); b) age from 16 to 78 years old of both genders; c) without serious cardiovascular disease, cerebrovascular disease, liver or kidney functional damage; d) psoriasis vulgaris patients should not be using systemic medication for the last 3 months but applying topical drugs; erythrodermic psoriasis patients should not be using systemic medication in the last 3 months but applying long-term topical emollients; arthropathic psoriasis patients should be using no drugs; and pustular psoriasis patients (palm and plantar only) should not be using systemic medication but undergoing ultraviolet A irradiation. Exclusion criteria were as follows: a) malignant skin tumor history; b) pregnant and lactating women; c) mental or neurological disease; d) immunocompromised patients.

According to the inclusion and exclusion criteria, a total of 157 cases were enrolled, and venous blood samples and clinical data were collected. The sample included: 65 cases of psoriasis vulgaris (active stage, 33 cases; stationary stage, 32 cases), 32 cases of erythrodermic psoriasis, 30 cases of arthropathic psoriasis, and 30 cases of pustular psoriasis (palm and plantar). Blood samples and data were also collected from 35 healthy controls (HCs) who had undergone a medical check-up at the Shanghai Skin Diseases Hospital from September 2013 to January 2015. Meanwhile, we selected seven skin specimens from the lesion and non-lesion areas of erythrodermic psoriasis cases. For the psoriasis vulgaris, pustular psoriasis and arthropathic psoriasis cases, we used the Psoriasis Area Severity Index (PASI), which cannot be used for erythrodermic psoriasis patients, as described previously (18).

The study protocol was approved by the ethics committee of the Shanghai Skin Diseases Hospital, and the study was conducted in accordance with the Declaration of Helsinki and international guidelines. All participants provided written informed consent.

### Isolation of human peripheral blood mononuclear cells

A total of 4-mL venous blood was drawn from HC and psoriasis patients, placed in a heparinized tube, and shaken well. Next, the samples were centrifuged at 729 *g* (4°C) for 5 min, and the resulting plasma was preserved. Then, 4 mL phosphate buffered saline (PBS) was added to the remaining blood and the sample was mixed. A pipette was used to add 4 mL Ficoll-hypaque lymphocyte separation medium (Hua Jing Technology Co., Ltd., China) to the samples, gently along the tube wall. The samples were centrifuged at 729 g (4°C) for 20 min. Then, a flat straw was used to remove the white blood cells from the middle layer. The cells were washed twice in PBS, and centrifuged at 729 *g* (4°C) for 5 min between each wash. The supernatant was removed and the cell pellet was resuspended in 1 mL PBS. Finally, a 10-µL sample of PBMCs was used for cell counting. A total of 1×10^6^ cells were taken for subsequent staining.

### Isolation of skin cells

Skin samples taken from the lesions and non-lesion areas of the skin of 7 erythrodermic psoriasis patients were cut into pieces. Cut samples were immersed in a digestive enzyme at 37°C for 2 h. The samples were washed twice with 1 mL RPMI-1640 medium (Gibco, USA), and centrifuged at 729 *g* (4°C) for 10 min between each wash. The supernatant was discarded, and the pellet was resuspended in 1 mL RPMI-1640 medium. After cell counting using a 10-µL sample, 1×10^6^ cells were taken for subsequent staining.

### Fluorescent antibody staining

A total of 100 µL of diluted cells from the psoriasis patients (peripheral blood and lesions from patients with erythrodermic psoriasis) and from the control group (HC peripheral blood and non-lesions from patients with erythrodermic psoriasis) were added to 11 separate 1.5 mL Eppendorf tubes. For both groups, one tube was used as a negative control, and to the other 7 tubes were added 2 µL mouse anti human FITC-labeled CD19, PE-labeled CD40, APC-labeled CD44, PE-labeled CD80, APC-labeled CD86, PE-labeled CD11b, and APC-labeled HLA-DR monoclonal antibodies (BD Biosciences, USA). A mixture of 2 µL FITC-labeled CD19, 2 µL PE-labeled CD40, and 2 µL APC-labeled CD44 was added to the ninth tube. In the tenth tube, 2 µL FITC-labeled CD19, 2 µL PE-labeled CD80, and 2 µL APC-labeled CD86 was added. In a final tube, 2 µL FITC-labeled CD19, 2 µL PE-labeled CD11b, and 2 µL APC-labeled HLA-DR was added. The samples were then placed at 4°C for 30 min in dark. After that, the samples were washed with PBS twice, and centrifuged at 729 *g* (4°C) for 5 min between washes. The supernatant was removed and the cell pellet was analyzed using an ACS Calibur flow cytometer (BD Biosciences, the process strictly complied the guidelines of the equipment).

### Statistical analysis

All statistical analyses were conducted using SPSS version 13.0 (SPSS Inc., USA). Data are reported as means±SD. Statistical significance was evaluated by independent sample *t*-test or one-way analysis of variance (ANOVA). Pearson’s correlation test was used to determine the correlation coefficient (r). A P-value of less than 0.05 was considered to be statistically significant (P<0.05)

## Results

### Baseline data

All 157 subjects were of Han nationality and were diagnosed with psoriasis. The disease duration ranged from one month to 20 years, with an average of 5.24±3.61 years, and there was no family history of psoriasis. The peripheral blood samples were divided into the following six groups: 1) psoriasis vulgaris at the active stage (n=33, 18 males and 15 females, aged 20 to 76 years, average of 41.57±4.99 years); 2) psoriasis vulgaris at the stationary stage (n=32, 16 males and 16 females, aged 22 to 71 years, average of 42.10±4.12 years); 3) erythrodermic psoriasis (n=32, 24 males and 8 females, aged 26 to 75 years, average of 49.25±4.23 years); 4) arthropathic psoriasis (n=30, 11 males and 19 females, aged 30 to 75 years, average of 52.53±4.28 years); 5) pustular psoriasis (palm and plantar; n=30, 12 males and 18 females, aged 20 to 78 years, average of 41.6±6.63 years); 6) HC (n=35, 22 males and 13 females, aged 18 to 75 years, average of 41.50±4.46 years). There were no significant differences with regards to gender and age among the six groups (P>0.05).

Of the 7 erythrodermic psoriasis patients selected for comparison of lesions and non-lesion areas, 5 were male and 2 were female (aged 39 to 79 years, average of 53.88±7.10 years).

### Proportion of CD19+ B cells

**Figure 1:**
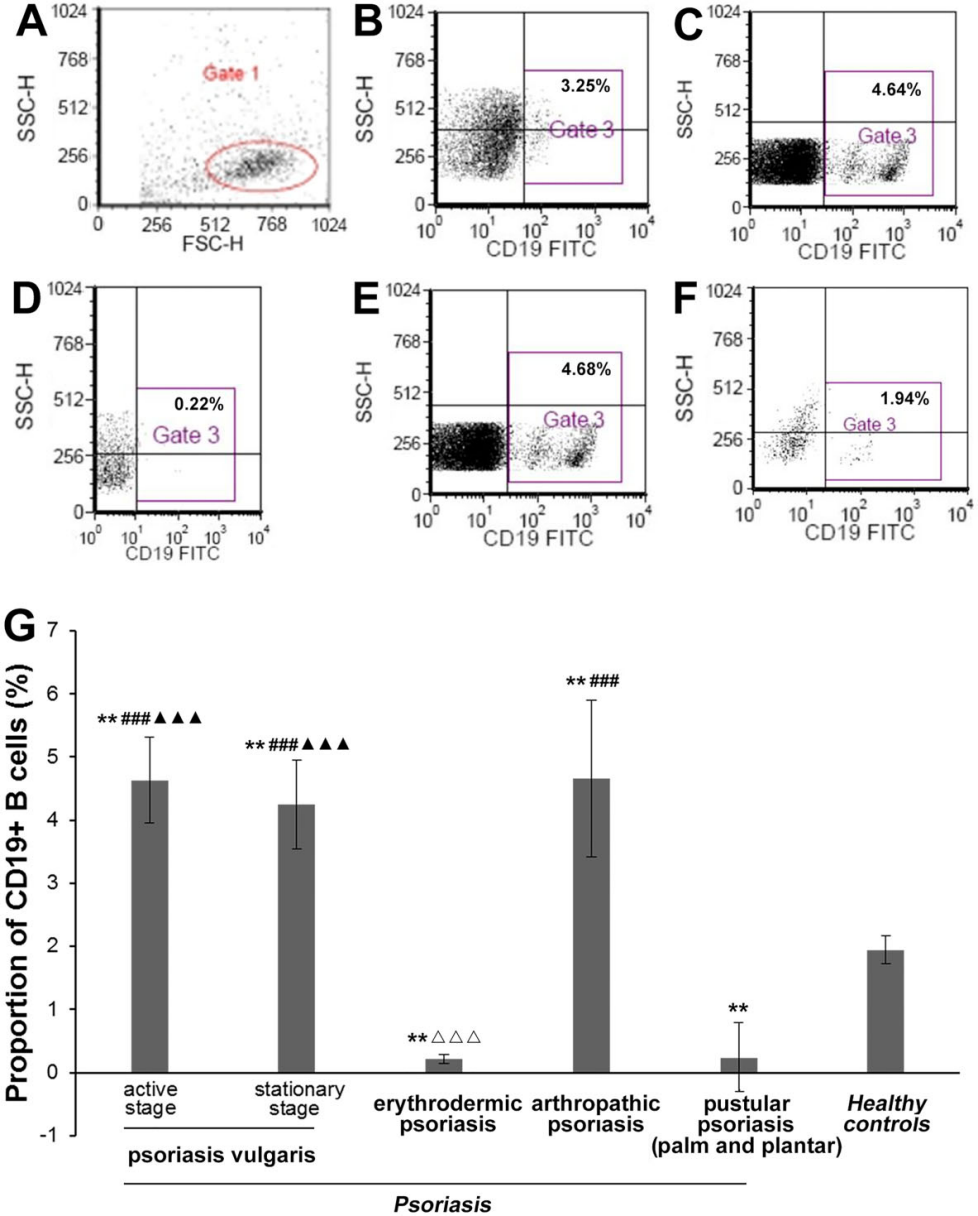
Proportion of CD19+ B cells in peripheral blood mononuclear cells (PBMCs) from patients with psoriasis and healthy controls, determined by flow cytometry. *A*, total lymphocytes from PBMCs (Gate 1). Proportion of CD19+ B cells (Gate 3) from PBMCs in the healthy controls (*B*), or patients with psoriasis vulgaris (*C*), erythrodermic psoriasis (*D*), arthropathic psoriasis (*E*), and pustular psoriasis (palm and plantar) (*F*). *G*, proportion of CD19+ B cells are shown as means± SD (n=33 for psoriasis vulgaris at the active stage; n=32 for psoriasis vulgaris at the stationary stage; n=32 for erythrodermic psoriasis; n=30 for arthropathic psoriasis; n=30 for pustular psoriasis (palm and plantar); n=35 for healthy controls). *P<0.05, **P<0.01 *vs* healthy controls; ^###^P<0.001 *vs* pustular psoriasis (palm and plantar); ^ΔΔΔ^P*<*0.001 *vs* arthropathic psoriasis; ^▾▾▾^P*<*0.001 *vs* erythrodermic psoriasis (one-way analysis of variance).

The proportion of CD19+ B cells in PBMCs from patients with psoriasis vulgaris (at both the active and stationary stage) and arthropathic psoriasis was higher compared with HCs (both P<0.01). However, the proportion of CD19+ B cells in PBMCs was lower in patients with erythrodermic psoriasis and pustular psoriasis compared with HCs (both P<0.01). Moreover, the proportion of CD19+ B cells in PBMCs from patients with psoriasis vulgaris was significantly higher than in the patients with erythrodermic psoriasis and pustular psoriasis (both P<0.001). The proportion of CD19+ B cells in PBMCs from patients with arthropathic psoriasis was also significantly higher than in the patients with erythrodermic psoriasis (P<0.001; Figure 1).

### Proportion of CD19+ B cells with the cell surface activation markers

**Figure 2:**
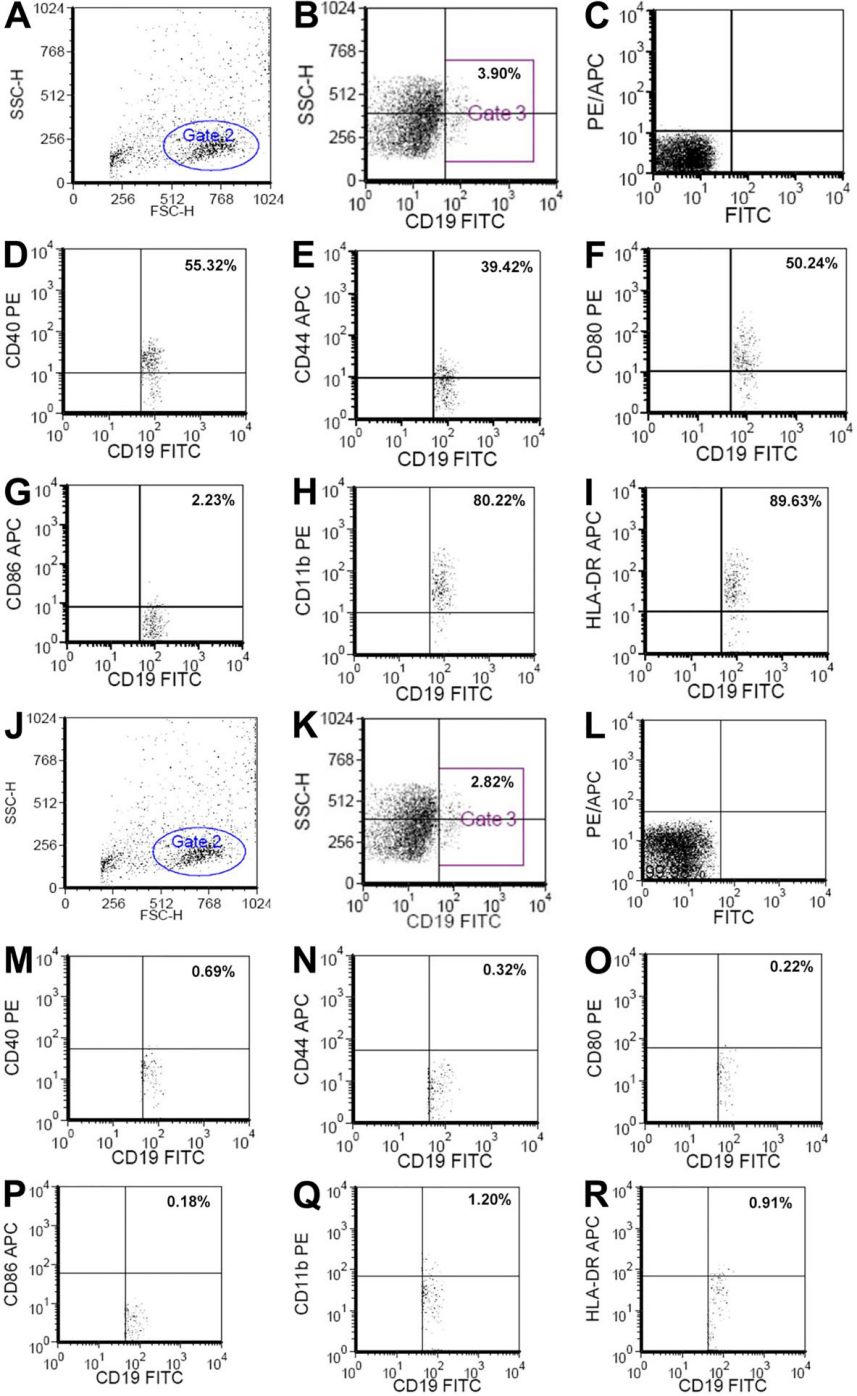
Representative images showing the proportion of B cell surface activation markers in peripheral blood mononuclear cells (PBMCs) from one patient with psoriasis vulgaris at the active stage (*A*-*I*) and one healthy control (*J*-*R*) determined by flow cytometry. *A*, total lymphocytes from PBMCs (Gate 2). *B*, proportion of CD19+ B cells from PBMCs (Gate 3). *C*, negative control (FITC and PE/APC). Proportion of CD19+CD40+ (*D*), CD19+CD44+ (*E*), CD19+CD80+ (*F*), CD19+CD86+ (*G*), CD19+CD11b+ (*H*), and CD19+HLA-DR+ (*I*) B cells from PBMCs. *J*, total lymphocytes from PBMCs (Gate 2). *K*, proportion of CD19+ B cells from PBMCs (Gate 3). *L*, negative control (FITC and PE/APC). Proportion of CD19+CD40+ (*M*), CD19+CD44+ (*N*), CD19+CD80+ (*O*), CD19+CD86+ (*P*), CD19+CD11b+ (*Q*), and CD19+HLA-DR+ (*R*) B cells from PBMCs.

**Figure 3:**
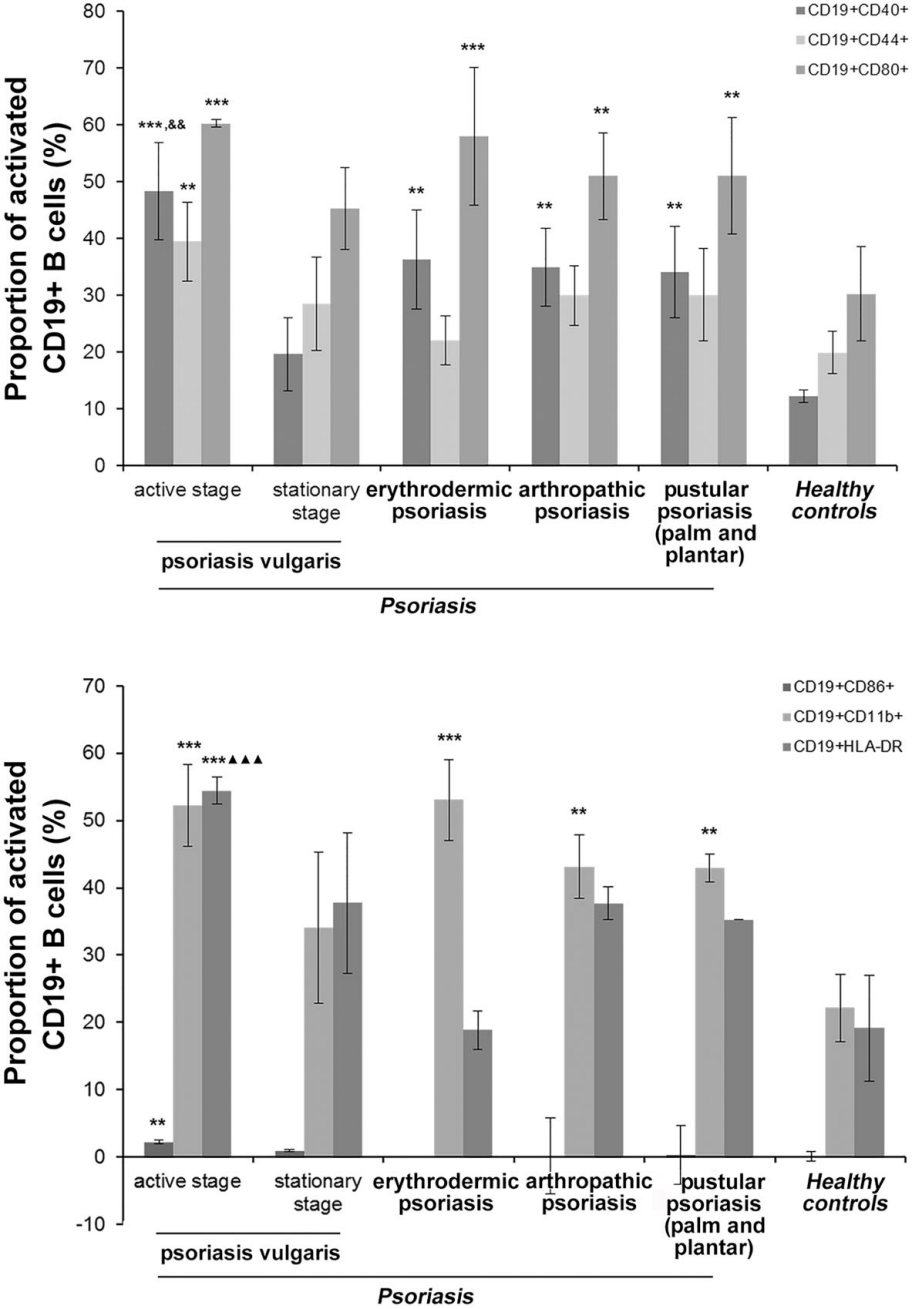
Proportion of CD19+ B cells surface activation markers in peripheral blood mononuclear cells, determined by flow cytometry. **P*<*0.01, ***P*<*0.001 *vs* healthy controls; ^&&^P<0.05 *vs* psoriasis vulgaris at the stationary stage; ^▾▾^P<0.01, ^▾▾▾^P<0.001 *vs* erythrodermic psoriasis. Data are reported as means±SD (n=33 for psoriasis vulgaris at the active stage; n=32 for psoriasis vulgaris at the stationary stage; n=32 for erythrodermic psoriasis; n=30 for arthropathic psoriasis; n=30 for pustular psoriasis (palm and plantar); n=35 for healthy controls). Statistical analysis was performed with one-way analysis of variance.

Representative flow cytometry images showing the proportion of CD19+CD40+, CD19+CD44+, CD19+CD80, CD19+CD86+, CD19+CD11b+, and CD19+HLA-DR+ B cells in PBMCs from a patient with psoriasis vulgaris at the active stage and a HC are shown in Figure 2. The proportions of these same B cell markers in PBMCs from the patients with the different psoriasis subtypes and HCs are shown in Figure 3.

The proportion of CD19+CD40+ B cells in PBMCs from patients with psoriasis vulgaris at the active stage (P<0.001), but not the stationary stage, and all other different psoriasis subtypes (all P<0.01) was upregulated compared with HCs. Moreover, the proportion of CD19+CD40+ B cells in PBMCs from patients with psoriasis vulgaris at the active stage was higher than psoriasis vulgaris at the stationary stage (P*<*0.01). The ratio of CD19+CD44+ B cells in PBMCs from patients with psoriasis vulgaris at the active stage was upregulated compared with HCs (P<0.01). The proportion of CD19+CD80+ B cells in PBMCs from patients with psoriasis vulgaris at the active stage (P<0.001), but not the stationary stage, and all other different psoriasis subtypes (all P<0.01) was upregulated compared with HCs. The proportion of CD19+CD86+ B cells in PBMCs was only increased in patients with psoriasis vulgaris at the active stage compared to HCs (P*<*0.01). The proportion of CD19+CD11b+ B cells in PBMCs from patients with psoriasis vulgaris at the active stage and erythrodermic psoriasis (P<0.001), but not the stationary stage, and other different psoriasis (all P<0.01) was upregulated compared with HCs. Finally, the proportion of CD19+HLA-DR+ B cells in PBMCs from patients with psoriasis vulgaris at the active stage was significantly upregulated compared with patients with erythrodermic psoriasis and HCs (P*<*0.001; Figure 3).

### CD19+ B cell subsets in skin lesions and non-lesions of patients with erythrodermic psoriasis

**Figure 4:**
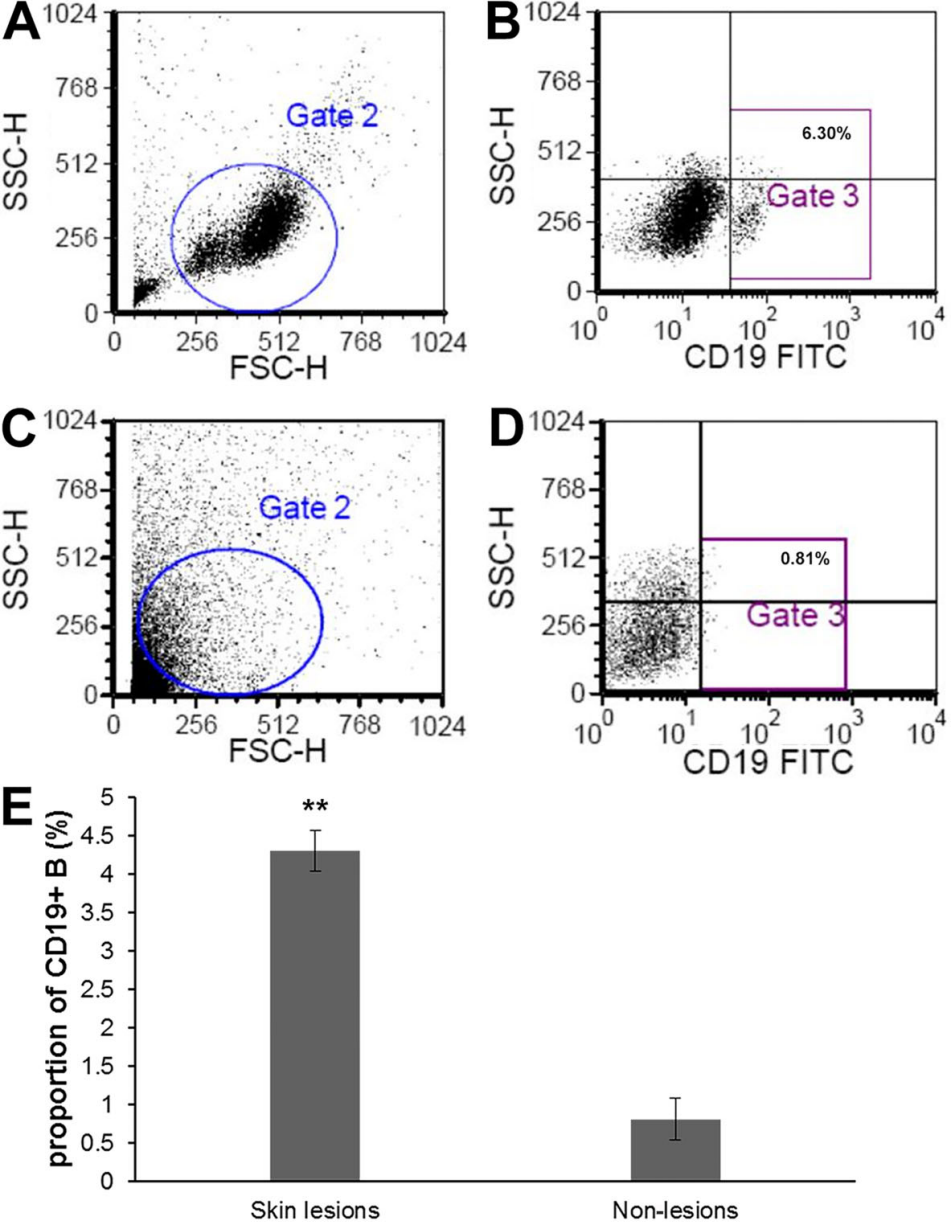
*A*, total lymphocytes and *B*, proportion of CD19+ B cells from the skin lesion of a patient with erythrodermic psoriasis (Gate 2 and 3, respectively). *C*, total lymphocytes and *D*, proportion of CD19+ B cells from a non-lesion area of a patient with erythrodermic psoriasis (Gate 2 and 3, respectively). *E*, proportion of CD19+ B cells from skin lesions and non-lesion areas of erythrodermic psoriasis patients are shown as means±SD (n=7 for skin lesions; n=7 for non-lesions). **P<0.001 *vs* non-lesions (one-way analysis of variance).

**Figure 5:**
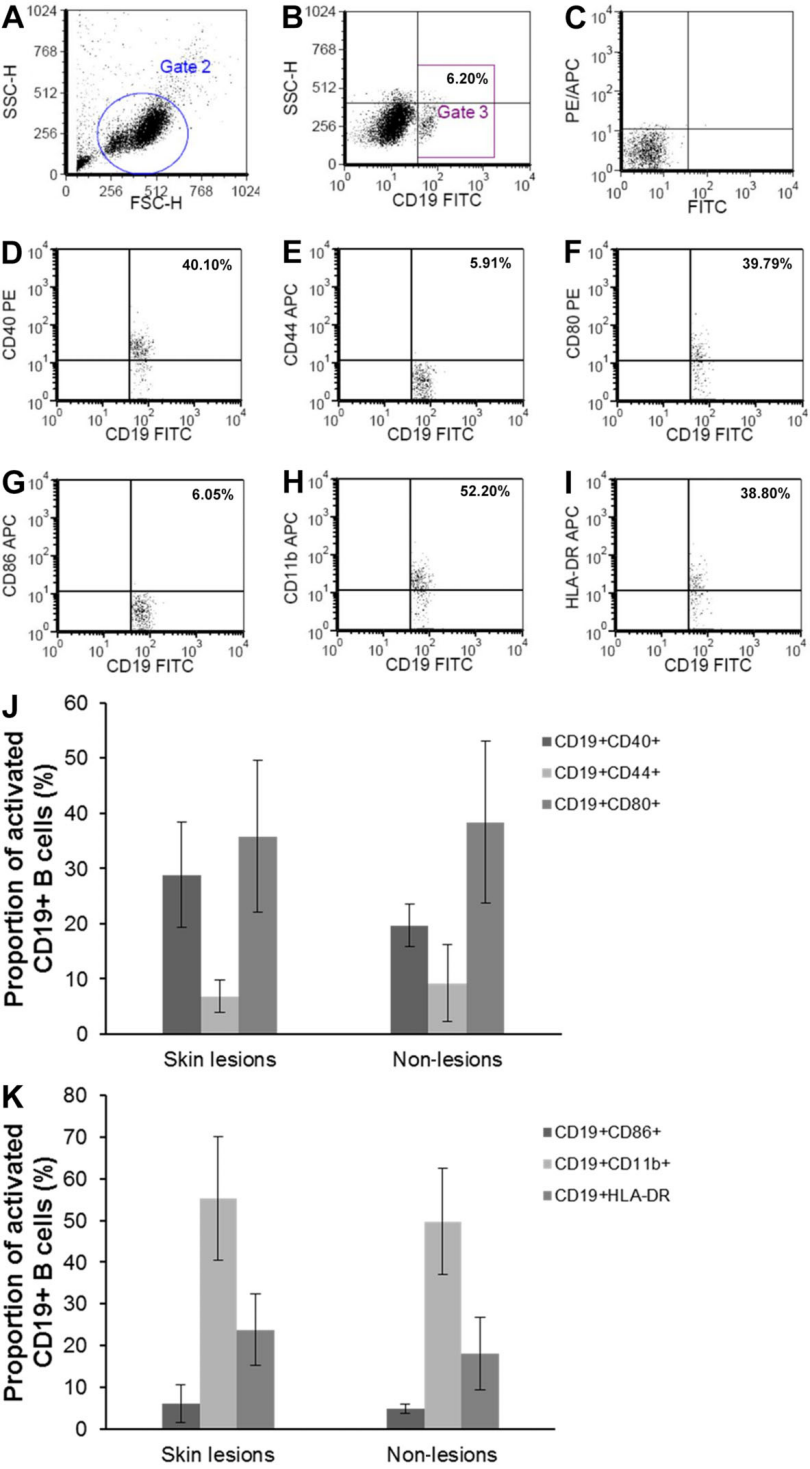
*A*, total lymphocytes (Gate 2); *B*, proportion of CD19+ B cells (Gate 3); and *C*, negative control of FITC and PE/APC from the skin lesion of a patient with erythrodermic psoriasis. The proportion of CD19+CD40+ (*D*), CD19+CD44+ (*E*), CD19+CD80+ (*F*), CD19+CD86+ (*G*), CD19+CD11b+ (*H*), and CD19+HLA-DR+ (*I*) B cells from the skin lesion of the patient are also represented. The proportions of CD19+CD40+, CD19+CD44+, and CD19+CD80+ (*J*), and CD19+CD86+, CD19+CD11b+, and CD19+HLA-DR+ (*K*) B cells from the skin lesions and non-lesion areas of the patient are shown as means±SD (n=7 for both skin lesions and for non-lesions).

The proportion of CD19+ B cells from skin lesions of patients with erythrodermic psoriasis was 4.30±1.10%, which was significantly higher than in non-lesions (0.81%±0.27%; P*<*0.001; Figures 4 and 5).

As the proportion of CD19+ B cells from non-lesions of erythrodermic psoriasis was quite low (<1%), we used the data obtained from flow cytometry for the expression of CD19+ B cell surface activation markers, which are shown in Figure 5. No significant differences in the CD19+ B cell subsets between skin lesions and non-lesions were found.

### Correlation between CD19+ B cell subsets and psoriasis severity

For the psoriasis vulgaris, pustular psoriasis, and arthropathic psoriasis groups, the PASI scores ranged from 1.23 to 16.69 (average 9.68±1.26).

The proportions of CD19+ B cells were positively correlated with the PASI scores of psoriasis vulgaris at the active and stationary stage (active stage: r=0.871, P=0.0013; stationary stage: r=0.683, P=0.0027) and PASI scores of arthropathic psoriasis (r=0.692, P=0.0026). However, the proportion of CD19+ B cells was negatively correlated with the PASI score of pustular psoriasis (r=-0.569, P*=*0.0035).

The ratios of CD19+CD40+, CD19+CD80+, and CD19+CD11b+ B cells were positively correlated with the PASI scores of psoriasis vulgaris at the active stage, arthropathic psoriasis, and pustular psoriasis (r=0.452, r=0.712, r=0.320, and P=0.0037, P*=*0.0025, and P*=*0.0044, respectively). The ratios of CD19+CD44+, CD19+CD86+, and CD19+HLA-DR+ B cells were also positively correlated with the PASI scores of psoriasis vulgaris at the active stage (r=0.640, r=0.914, r=0.334, and P*=*0.0029, P*=*0.0011 and P*=*0.0043, respectively).

## Discussion

Psoriasis is an inflammatory cutaneous disorder, however, the contribution of B cells to its pathogenesis remains unclear. In this study, we investigated the ratios of CD19+ B cells in PBMCs of lesions and non-lesion areas of psoriasis patients. We found altered proportions of CD19+ B cell subsets in PBMCs from the different clinical subtypes of psoriasis. Moreover, the proportion of CD19+ B cells found in skin lesions of patients with erythrodermic psoriasis was higher than that seen in non-lesions. Finally, the PASI score was positively correlated with CD19+ B cell ratios in psoriasis vulgaris at the active and stationary stage and arthropathic psoriasis, but negatively correlated in pustular psoriasis. Together, these results suggest that CD19+ B cells are important in the pathogenesis of psoriasis.

Our results are in agreement with previous studies that indicate that B cells, such as regulatory B10 cells, are important in mouse models of psoriasis (19). We showed the proportion of CD19+ B cells in PBMCs from patients with psoriasis vulgaris at both the active and stationary stage and arthropathic psoriasis was higher than in controls, indicating that CD19+ B cell levels increase during the early pathogenesis of psoriasis (20–22). On the other hand, the proportion of CD19+ B cells in PBMCs from erythrodermic psoriasis patients was lower than that in HC; however, B cell infiltration was still observed in the skin lesions in these patients. This result suggests that CD19+ B cells in the peripheral blood are depleted in the later stages of psoriasis, and most CD19+ B cells have migrated to the lesion areas.

In this study, the PASI scores of psoriasis vulgaris at the active and stationary stage and arthropathic psoriasis were positively correlated with the ratio of CD19+ B cells in PBMCs. However, the PASI scores of pustular psoriasis were negatively correlated with the ratio of CD19+ B cells in PBMCs. Moreover, the ratios of CD19+ B cells were altered in patients with different clinical subtypes of psoriasis. For example, a low ratio of CD19+ B cells was observed in erythrodermic psoriasis patients, suggesting a potential correlation between CD19+ B cell levels and psoriasis classification.

Our observation that the ratio of CD19+CD40+ B cells was increased in psoriasis vulgaris at the active stage was similar to a previous study that showed an increase in CD40+ cells, some of which were dendritic in shape, in the dermal infiltrate of lesions from patients with psoriasis vulgaris compared with normal skin (23). Our results indicated that CD44 was significantly elevated in PBMCs in patients with psoriasis vulgaris. Although it was previously shown that there was an increased proportion of CD44+ inflammatory and endothelial cells in PBMCs in psoriasis vulgaris skin lesions than in non-lesion areas (24), we found no difference in the ratio of CD19+CD44+ B cells between erythrodermic psoriasis skin lesions and non-lesions. We also found an elevated ratio of CD19+CD80+ B cells in the PBMCs from all subtypes of psoriasis in this study (except for psoriasis vulgaris at the stationary stage). However, the ratio of CD19+CD86+ B cells was only upregulated in PBMCs in psoriasis vulgaris at the active stage. Therefore, our results confirm that CD40, CD44, CD80, and CD86 are likely important in the pathogenesis of psoriasis, especially at the active stage.

CD11b expression on leukocytes has been suggested as a monitoring index for pustular psoriasis activity (16). Indeed, we found that the ratio of CD19+CD11b+ B cells in PBMCs was high in patients with all psoriasis subtypes (except for psoriasis vulgaris at the stationary stage), including pustular psoriasis. On the other hand, the ratio of CD19+HLA-DR+ B cells was only high in PBMCs from psoriasis vulgaris at the active stage. HLA-DR may play a role in antigen presentation to activate T cells during the early stages of psoriasis pathogenesis. Moreover, although the peripheral blood CD19+ B cells may have been exhausted in erythrodermic psoriasis patients, we still observed a higher proportion of CD19+CD40+, CD19+CD80+, and CD19+CD11b+ B cells in the PBMCs compared to HCs, indicating that some of the B cells were still active. However, the ratio of CD19+HLA-DR+ B cells was similar to that observed in HCs, which indicates that in the later phase of the disease, B cells are no longer functioning as antigen presenting cells, but as regulatory cells to control the occurrence and development of psoriasis.

From the proportion of CD19+ B cell surface activation markers in the above four types of psoriasis vulgaris, we speculate that the expressions of B cell surface activation markers are different in various types of psoriasis, and activated B cells play distinct roles in the different stages of disease. For example, at later pathogenic stages of psoriasis, B cells become regulatory cells rather than antigen presenting cells. The observation that different subsets of B cells are activated at different pathological stages of psoriasis, or in different clinical subtypes, is similar to that seen in T cells. Inaoki et al. (25) showed different T cell subsets are activated in psoriasis, and that L-selectin expression levels on CD4+ T cells correlated with psoriasis severity. Similarly, O'Daly et al. (21) showed that T lymphocyte subsets vary according to PASI scores. These particular B-lymphocyte subsets may be potential indicators of disease severity.

There were some limitations in this study. In particular, we have not investigated whether these CD19+ B cell subsets change upon psoriasis treatment. In the future, we will detect the ratios of CD19+ B cells before and after treatment, similar to the study on CD3+CD56+ NK T cells performed by Koreck et al. (26). However, understanding the exact mechanisms of how B cells contribute to the pathogenesis of psoriasis, and the importance of these various cell surface markers, requires further investigation.

In summary, the different ratios of CD19+ B cell subsets in various types of psoriasis indicate that these cells are potential indexes for clinical psoriasis classification. Moreover, in addition to T cells, B cell activation may be important at different pathological stages of psoriasis.
